# 

**Published:** 1785

**Authors:** 


### LONDON MEDICAL JOURNAL.

---

VOLUME THE SIXTH.

FOR THE YEAR 1785.

---

Surgeon Gen'l's Office. LIBRARY. 63445

LONDON:

PRINTED for the EDITOR,

And fold by JOSEPH JOHNSON, London;
CHARLES ELLIOT, Edinburgh;
And P. BYRNE, Dublin.
M,DCC,LXXXV.

### ADVERTISEMENT.

IN the future arrangement of this work, no particular ſection will be ſet apart for Medical Intelligence. The ſame diligence, however, will be exerted, as heretofore, in procuring and publiſhing early accounts of new and uſeful obſervations in Phyſic and Medical Philoſophy; but theſe, inſtead of forming part of a ſeparate ſection, as in the former volumes, will be given under the head of Eſſays and Obſervations, if communicated by correſpondents; or of that of Books, if collected from Journals or other printed works.

### CATALOGUE.

1. AN Effay on the ufefulnefs of Chemiftry, and its application to the various occafions of life. Tranflated from the original of Sir *Torbern Bergman*, Profeffor of Chemiftry at Upfal, &c. 8vo. *Murray*, London, 1783. 163 pages.

2. Some Thoughts on the relaxation of human bodies; and on the misapplication of the Bark in that and some other cases. 8vo. *Nicoll*, London, 1783. 101 pages.

3. *Johannis Brunonis*, M. D. de Medicina Prælectoris, Societatis Medicæ Præsidarii, Antiquariorum apud Scotos ab Epistolis latinis, Elementa Medicinæ. Editio altera plurimum emendata, et integrum demum opus exhibens. 8vo. Edinburgi. Tom. 2. 1784. Tom. I. p. 200. Tom. II. p. 227.

4. A Treatise on Struma or Scrophula, commonly called the King's Evil; in which the impropriety of considering it as an hereditary disease is pointed out; more rational causes are assigned; and a successful method of treatment is recommended. By *Thomas White*, Surgeon to the London Dispensary. 8vo. *Murray*, London, 1784. 110 pages.

5. A Treatise on Cancers, with a new and successful method of operating, particularly in Cancers of the Breast and Testis, whereby pain is considerably diminished, the cure or healing of the part greatly accelerated, and deformity prevented. By *Henry Fearon*, of the Company of Surgeons, and Surgeon to the Surrey Dispensary.

penſary. 8vo. *Johnſon*, London, 1784. 77 pages.

6. Obſervations on the Animal Oeconomy, and on the cauſes and cure of Diſeaſes. By *John Gardiner*, M. D. Preſident of the Royal College of Phyſicians, and Fellow of the Royal Society of Edinburgh. 8vo. *Creech*, Edinburgh, 1784. 458 pages.

7. Symptomatology. By *John Berkenhout*, M. D. 8vo. *Baldwin*, London, 1784.

8. Some Conſiderations on the different ways of removing confined and infectious air; and the means adopted, with remarks on the contagion in Maidſtone gaol. By *Thomas Day*, Surgeon. To which is added, an appendix, containing ſome experiments on ventilating ſmall ſitting rooms, and preventing chimnies from ſmoking. 8vo. *Blake*, Maidſtone, 1784.

9. Medical Caſes, with occaſional remarks. By *R. W. Stack*, of Bath, M. D. 8vo. *Johnſon*, London, 1784.

10. A Treatiſe on the influence of the Moon in fevers. By *Francis Balfour*, M. D. Surgeon in the ſervice of the Eaſt-India Company. 8vo. *Elliot*, Edinburgh, 1785.

11. Oratio coram Societate Phyſica, die quo primum ad Aedes novas dedicandas convenit, quam quam habuit *Thomas Addis Emmet*, M. D. ejus nec non Societatum Reg. Med. Nat. Hift. et Specul. Præfes annuus. 8vo. *Elliot*, Edinburgh, 1785.

12. An Effay on the Jaundice, in which the propriety of ufing the Bath waters in that difeafe, and in fome particular affections of the Liver is confidered. By *William Corp*, M. D. Member of the Royal Medical Society at Edinburgh, and Phyfician to the Pauper Charity at Bath. 8vo. *Cruttwell*, Bath, 1784.

13. Report of Dr. *Benjamin Franklin*, and other Commiffioners, charged by the King of France, with the examination of the Animal Magnetifm, as now practifed at Paris. Tranflated from the French, with an Hiftorical Introduction. 8vo. *Johnfon*, London, 1785. 108 pages.

14. An Effay on the Medical Character, with a view to define it. To which is fubjoined, Medical Commentaries and Obfervations, adapted to feveral cafes of indifpofed health. By *Robert Bath*. 8vo. *Laidler*, London, 1785. 209 pages.

15. Differtatio Medica Inauguralis de Mirabile, quæ caput inter et partes Generatoni dicatas intercedit, Sympathia. Auctore *Jano Peterfen*

*terfen Michell*, Amftelædamenfi. 4to. Lugd. Batav. 1781. p. 74.

16. *L. M. A. Caldanii* in Patavino Lycæo Medicinæ Theoreticæ et Anatomiæ Profefforis publici primarii, &c. Inftitutiones Phyfiologicæ et Pathologicæ. Edidit, præfatus eft, indicemque addidit *Edvardus Sandifort*, Medicinæ, Anatomes et Chirurgiæ in academia Batava, quæ Leidæ eft, Profeffor, &c. 8vo. Tom. 2. 1784.

17. *Antonii Scarpa*, dudum in Mutinenfi, nunc in R. Ticin. Archigymn. Anat. et Chirurg. Operat. Profeff. Oratio de promovendis anatomicarum adminiftrationum rationibus ad tyrones habita in Audit. Magn. Archigymn. cum. trad. Anat. munus pub. aufpicaretur VI. Kal. Decemb. ann. 1783. 4to. Pavia.

18. Rapport de l'un des Commiffaires chargés par le Roi de l'examen du Magnetifme Animal. *i. e.* Report of one of the Committee appointed by the King to inquire into Animal Magnetifm. 4to. Paris, 1784. 54 pages.

Two Committees have been appointed at Paris, by Royal authority, to make inquiries concerning Animal Magnetifm; of the Report of one of thefe, which confifted of Members of the Faculty of Phyfic and Academy of Sciences, we we gave an account in a former volume *. The other Committee was composed of Members of the Royal Medical Society, of whose Report †, though written with great learning and judgment, we thought it superfluous to give any particular account, as it agreed with the former in all the leading points of the subject. The report now before us, which may be considered as a Protest, is by M. Ant. de Jussieu, of the Royal Medical Society, who seems to be a well-meaning man, but a little too credulous.

19. Lettre d'un Bordelois au Pere Hervier en reponse à celle que ce savant a ecrite aux Bordelois a l'occasion du Magnetisme Animal. *i. e.* Letter from an inhabitant of Bourdeaux to Father Hervier, in answer to one which the learned Father addressed to the inhabitants of Bourdeaux concerning Animal Magnetism. 8vo. Bourdeaux, 1784. 16 pages.

20. Lettre d'un Medecin de la Faculté de Paris à M. Court de Gibelin, en reponse à celle que ce savant a addressé à ses souscripteurs, & dans laquelle il fait un eloge triomphant du Magnetisme Animal. *i. e.* Letter from a Phy-

* Vol. V. p. 266.

† Ibid. p. 334.

ſician of the Faculty of Paris to M. Court de Gibelin in anſwer to one addreſſed by that learned writer to his ſubſcribers, and in which he delivers a triumphant eulogium on Animal Magnetiſm. 8vo. Bourdeaux, 1784. 67 pages.

21. Lettre a M. Deſlon, Medecin, &c. 8vo. Paris, 1784. 27 pages.

This Epiſtle, the author of which is Count Fontette Sommery, is in favour of Animal Magnetiſm.

22. Lettre de Figaro au Comte Alma Viva ſur la criſe du Magnetiſme Animal; avec des details propres a fixer enfin l'opinion ſur l'inutilité de cette decouverte. *i. e.* Letter from Figaro to Count Alma Viva on the criſis of Animal Magnetiſm; with particulars calculated to fix the opinion of the public concerning the inutility of that diſcovery. 8vo. Paris, 1784. 45 pages.

This is a piece of Pariſian wit, at the expence of M. Meſmer and Animal Magnetiſm.

23. L'antimagnetiſme, ou origine, progrés, decadence, renouvellement et refutation du Magnetiſme Animal. *i. e.* Anti Magnetiſm, or the origin, progreſs, fall, revival, and refutation of Animal Magnetiſm. 8vo. Paris, 1784. 252 pages.

 24. Anti-

24. Antidotarium Collegii Medici Bononienſis editum anno 1783. Editio noviſſima in qua locupletiſſimus adjectus eſt index virium ac uſuum medicamentorum. 4to. Venice, 1783. p. 240.

25. Bemerkungen uber einige einfache und Zuſammengeſetzte Arzneymittel. *i. e.* Obſervations on ſeveral ſimple and compound Medicines, by *Conrad Monch*, Aſſeſſor of the College of Phyſic, and Apothecary at Caſſel. 8vo. Frankfort, 1781.

26. Critiſche Nachrichten von kleinen Mediniſchen ſchriften, &c. *i. e.* Critical Account of the Medical Diſſertations publiſhed in German and other Univerſities in the year 1780. By *Chriſtian Godfrey Grunner*, Profeſſor of Phyſic at Jena, &c. Vol. I. 8vo. Leipſic, 1783.

The Univerſities, out of Germany, to which the author extends his review in this firſt volume, are Leyden, Utrecht, Upſal, Lund, Copenhagen, and Kiel.

27. Memoires de la Société de Sciences Phyſiques de Lauſanne. *i. e.* Memoirs of the Society of Phyſical Sciences at Lauſanne. Vol. I. for the year 1783. 4to. Lauſanne, 1784. 322 pages.

28. Dizionario di Chimica, &c. *i. e.* Dictionary of Chemiſtry. Tranſlated from the French of *P. J. Macquer*, with additions and notes, by *J. Ant. Scopoli.* 8vo. Pavia, 1783.

We have not yet ſeen this tranſlation of Macquer's Dictionary, but it is ſaid to be very judiciouſly executed. Only five volumes of it are as yet publiſhed. The fifth volume cloſes with the article Nitre.

29. Des Maladies des Creoles en Europe, avec la Maniere de les traiter; et des obſervations ſur celles des gens de mer, et ſur quelques autres plus frequemment obſervées dans les climats chauds. *i. e.* On the Diſeaſes of Creoles in Europe, with the manner of treating them; and remarks on thoſe of ſeafaring people, and on ſome others moſt frequently obſerved in hot climates. By *J. J. de Gardane*, Doctor Regent of the Faculty of Phyſic at Paris, &c. 8vo. Paris, 1784. 228 pages.

30. Memoires et Obſervations de Chimie, *i. e.* Chemical Eſſays and Obſervations. By *M. de Fourcroy*, M. D. of the Faculty of Phyſic at Paris, &c. intended as a ſequel of the Elements of Chemiſtry * publiſhed by the author in 1782. 8vo. Paris, 1784. p. 447.

* See vol. 4. p. 332.

31. Pofitiones Chemico-medicæ de aere vitali, feu dephlogifticato, tanquam novo fanitatis præfidio, ab auctore *Alexandro Poulle.* 12mo. Montpellier, 1784. p. 84.

32. *Antonii Michelitz* Phil. & Med. Doct. &c. Difquifitio Phyfiologica caufarum refpirationis. 8vo. Prague, 1783. p. 72.

33. *Petri Camper*, M. D. &c. Obfervationes circa mutationes quas fubeunt calculi in vefica, ex Belgico fermone in Latinum tranflatæ a *Jofepho Szombathy*, M. D. 4to. Peft, 1784. p. 16.

34. De præcipuis morborum mutationibus et converfionibus Tentamen medicum Authore *A. C. Lorry*, D. M. P. Editionem poft authoris fata curante *J. N. Hallé*, D. M. P. 8vo. Paris, 1784.

35. Nouveaux Elemens de Matiere Medicale, &c. *i. e.* New Elements of the Materia Medica extracted from the public leffons of *M. de Lamure*, Senior Profeffor of Phyfic in the Univerfity of Montpellier. Collected and arranged by *M*——, M. D. 4to. Paris, 1784. 482 pages.

36. Precis Chimique fur les principes de la formation de l'acide Nitreux. *i. e.* Chemical Effay on the principles of the formation of the

 Nitrous

Nitrous Acid; being the work which obtained the premium offered by the Royal Society of Sciences at Copenhagen in 1776. By M. *Thouvenel*, M. D. Correſponding Member of the Royal College of Phyſicians at Nancy, &c. 4to. Copenhagen, 1784. p. 32.

37. Apparatus Medicaminum tam ſimplicium quam præparatorum et compoſitorum in praxeos adjumentum conſideratas. Volumen tertium auctore *Jo. Andrea Murray*, M. D. Equite ord. R. de Waſa, Conſ. R. aul. Prof. Med. et Bot. Ord. in Acad. R. Gotting, &c. 8vo. Gottingæ, 1784. p. 572.

38. Handbuch der praktiſchen arzneywiſſenſchaft zum gebrauch fur angehende Aerzte. *i. e.* Compendium of the Practice of Phyſic for the uſe of young Phyſicians. By *Samuel Gottlieb Vogel*, M. D. &c. Vol. I. 8vo. Stendal, 1781.

39. Memoires et Obſervations ſur un nouveau moyen de prevenir et d'éviter l'aveuglement qui a pour cauſe la Cataracte. *i. e.* Eſſays and Obſervations on a new method of preventing and avoiding the blindneſs occaſioned by a cataract. By M. *Marchan*, Oculiſt of the city of Nimes, &c. 8vo. Nimes, 1784. p. 24.

40. Ver-

40. Vermium Inteſtinalium preſertim Tæniæ humanæ brevis expoſitio. Auctore *Paulo Chriſtiano Friderico Wernero*, Med. Baccalaureo. 8vo. Lipſiæ, 1782, cum tabulis æneis.

41. Sylloge Selectiorum opuſculorum argumenti medico-practici, collegit et edidit *Ern. Godofr. Baldinger*, Phil. et Med. Doct. Ord. Med. Gottingens. Primar. et ſenior Med. Pract. P. O. &c. Vol. VI. 8vo. Gottingæ, 1784. p. 360.

42. Elenchus Fungorum. Conſcripſit *Aug. Jo. Georg. Carol. Batſch.* Philoſ. Doct. Accedunt Icones 57 Fungorum nonnullorum Agri Jenenſis, Secundum Naturam ab auctore depictæ, æri inciſæ et vivis coloribus fucatæ a *J. S. Capieux.* 4to. Halæ Magdeburgicæ, 1783.

43. *Bernhard Ramazzini* Abhandlung von den Krankheiten der Kunſtler und Handwerker, &c. *i. e. Bernard Ramazzini's* Treatiſe on the Diſeaſes of Tradeſmen, reviſed and corrected, with additions, by *J. C. Gottlieb Ackermann*, M. D. &c. 8vo. Stendal. 2 vols.

*Jo. G. Roederer* et *Car. G. Wagleri* Tractatus de Morbo mucoſo, denuo recuſus annexaque præfatione de Trichuridibus novo vermium genere, editus ab *Henrico Auguſto Wriſberg*,

Pro-

Professore Medico et Anatomico Goettingensi, cum tabulis æneis. 8vo. Goettingæ, 1783. p. 331.

44. Memoire sur l' Electricité Medicale, &c. *i. e.* An Essay on Medical Electricity, which obtained the prize given in August 1783, by the Royal Academy of Sciences, Belles Lettres and Arts at Rouen. By M. *Marat.* 8vo. Paris, 1784. p. 111.

44. Analyse de l'eau minerale de Fruges. *i. e.* Analysis of the Mineral Water of Fruges, by *Peter de Ribaucourt,* Apothecary at Abbeville. 8vo. Abbeville, 1783. p. 28.

45. *Philippi Conradi Fabricii,* M.D. Serenissimi Ducis Brunsvicensium et Luneburg. à consiliis aulicis, Med. Prof. Publ. Ordin. Facultatis Med. Helmstadiensis presidis, &c. Animadversiones varii argumenti medicas ex Scriptis ejusdem minoribus collegit notisque adjectis edidit *Georg. Rudolph. Lichtenstein,* Med. Prof. 4to. Pars I. Helmstadt, 1783. p. 140.

46. De Morbis Nervorum eorumque frequentissima ex abdomine origine. Auctore *Joanne Heineken,* M. D. 4to. Gottingæ, 1783. p. 70.

47. Insti-

47. Inſtitutionum Medicinæ practicæ Tomus primus. Auctore *Joſ. Batiſta Borſieri.* 4to. Milan, 1784.

48. Extrait de la Correſpondance de la Société Royale de Medecine relativement au Magnetiſme Animal. *i. e.* Extract of the Correſpondence of the Royal Medical Society relative to Animal Magnetiſm. By M. *Thouret.* 4to. Paris, 1785. p. 74.

49. Des Specifiques en Medecine. *i. e.* On Specifics in Phyſic. By M. *Gaſtellier*, M. D. Phyſician in ordinary to the Duke of Orleans, and Member of the Royal Medical Society at Paris, &c. 8vo. Paris, 1783. p. 163.

50. Traité d'Oſteologie. *i. e.* Treatiſe of Oſteology. By M. *Bertier*, Doctor Regent of the Faculty of Phyſic, and Member of the Royal Academy of Sciences. A new edition; to which are added, three Eſſays by M. *Heriſſant*, on different ſubjects of Oſteology. 8vo. Paris, 1783. 4 vols.

51. Diſſertatio Medica de Sputis. Auctore *Chriſtiano G. F. Webel*, M. B. 4to. Lipſiæ, 1783, p. 42.

52. Diſſertatio Medica de Anthropophago Bercano. Auctore *Franciſco Gulielmo Antonio Jacobo de Hatzfeld*, M. D. 4to. Jena, 1781. p. 28.

